# Predictive Factors and Management of Macular Edema after Retropupillary Iris-Claw Intraocular Lens Implantation in Aphakia: National Multicenter Audit—Report 2

**DOI:** 10.3390/jcm12020436

**Published:** 2023-01-05

**Authors:** Carolina Bernal-Morales, Manuel Javier Navarro-Angulo, Mariano Rodriguez-Maqueda, Daniel Velazquez-Villoria, Juan Manuel Cubero-Parra, Joaquín Marticorena, Adrián Hernández-Martínez, Miguel Ruiz-Miguel, Alfredo Adan, Diego Ruiz-Casas, Javier Zarranz-Ventura

**Affiliations:** 1 Institut Clínic d’Oftalmologia (ICOF), Hospital Clínic, 08028 Barcelona, Spain; 2 August Pi i Sunyer Biomedical Research Institute (IDIBAPS), 08036 Barcelona, Spain; 3 Hospital Universitario Vírgen del Rocío, 41013 Sevilla, Spain; 4 Hospital Povisa, 36211 Vigo, Spain; 5 Hospital La Arruzafa, 14012 Córdoba, Spain; 6 Instituto Oftalmológico La Esperanza, HM La Esperanza, 15705 Santiago de Compostela, Spain; 7 Servicio de Oftalmología, Complejo Hospitalario Universitario, 15006 A Coruña, Spain; 8 Hospital Universitario Valme, 41014 Sevilla, Spain; 9 Hospital Donostia, 20014 San Sebastián, Spain; 10 Servicio de Oftalmología, Hospital Ramon y Cajal, 28029 Madrid, Spain

**Keywords:** macular edema, retropupillary iris-claw, pars plana vitrectomy, aphakia

## Abstract

The aim of this multicenter, national clinical audit is to evaluate the predictive factors and management of postoperative macular edema (ME) after retropupillary iris-claw intraocular lens (RICI) implantation and pars plana vitrectomy (PPV). Preoperative, surgical and postoperative data were collected. Number and type of intravitreal injections (IT) administered (anti-VEGF or dexamethasone implant), visual acuity (VA), intraocular pressure (IOP) and central retinal thickness (CRT) assessed by OCT were collected at 1, 3, 6 and 12 months. From 325 eyes (325 patients), 11.7% (38/325) developed postoperative ME. Previous complicated cataract surgery with no capsular support was the only significant predictive factor for developing postoperative ME (OR 2.27, 95% CI 1.38–4.52, *p* = 0.02) after RICI implant. Mean time to ME development was 11.4 ± 10.7 weeks, and mean CRT peaked at 3 months follow-up. Different treatment options were non-steroidal anti-inflammatory (NSAIDs) drops (31.6%, 12/38), dexamethasone (DEX) implant (50%, 19/38), anti-VEGF (7.9%, 3/38) or combined IT (10.5%, 4/38). Cumulative probability of ME resolution was higher in the group treated with IT than in the group treated with topical NSAIDs (85.2% vs. 58.3%, *p* = 0.9). Performing RICI implantation after complicated cataract surgery is a risk factor for the development of postoperative ME. DEX implants may be an effective treatment for postoperative ME in these cases.

## 1. Introduction

Retropupillary Iris-Claw Intraocular lens (RICI) implantation has been described as an effective surgical option to solve aphakia after complicated cataract surgery or intraocular lens (IOL) luxation [1,2,3,4] Nevertheless, this technique presents a series of complications among which the development of postoperative macular edema (ME) has been defined as one of the main causes of visual loss after RICI implantation [4,5,6,7]. Postoperative ME has already been described after uneventful cataract surgery or pars plana vitrectomy, even though continuous improvements in surgical techniques and transformation to small incision surgery have significantly decreased its incidence in the last few years [8,9,10,11,12,13].

To date, postoperative ME is known to have a multifactorial pathogenesis and diagnosis is mainly based on fundus examination and spectral-domain optical coherence tomography (SD-OCT), leaving behind previous diagnostic tools such as fundus fluorescein angiography (FFA) [9]. Since first described in 1953, postoperative ME has been associated with some risk factors such as uveitis, diabetes mellitus (DM), the presence of an epiretinal membrane, postoperative inflammation and posterior capsule rupture with vitreous loss [9,14,15,16] Therefore, it is reasonable that ayes with RICI implantation after complicated cataract surgery or IOL luxation are more at risk of developing postoperative ME. However, little is known about the real incidence of this complication in cases of RICI implantation and figures differ in the published literature [2,3,6].

The management of postoperative ME is not standardized and usually a staggered therapy is followed. First-line treatment is composed of a decrescent regimen of topical non-steroidal anti-inflammatory drops (NSAIDs) associated or not with topical steroids or off-label oral acetazolamide. Second-line therapies consist of a wide range of off-label treatments which include the injection of periocular steroids such as triamcinolone in the sub-Tenon’s or retrobulbar space, the intravitreal injection of steroids (triamcinolone or slow-release dexamethasone implants) or anti-vascular endothelial growth factor (anti-VEGF) injections, with variable outcomes as reported by previous authors [15,16,17,18,19,20].

The aim of the present study is to analyze possible predictive factors for the development of postoperative ME in patients with previous complicated cataract surgery with no capsular support or IOL luxation who underwent RICI implantation and PPV in a large, multicentric, national, routine clinical care audit. Secondary aims are to further explore the clinical outcomes and safety of different types of treatment employed in these cases of postoperative ME after RICI implantation.

## 2. Materials and Methods

Multicenter, retrospective, descriptive, real-world clinical practice review of eyes which developed postoperative ME after RICI implantation and PPV in eyes with previous complicated cataract surgery with no capsular support or IOL luxation in a 5-year period (2014–2019) [4]. All relevant data were systematically collected in a comprehensive spreadsheet distributed to the 14 participating centers and merged in a centralized database in the coordinating center for analysis. The review board at the coordinator center (CEIM, Hospital Clínic of Barcelona) approved this study (study code HCB/2018/0182).

**Figure 1:**
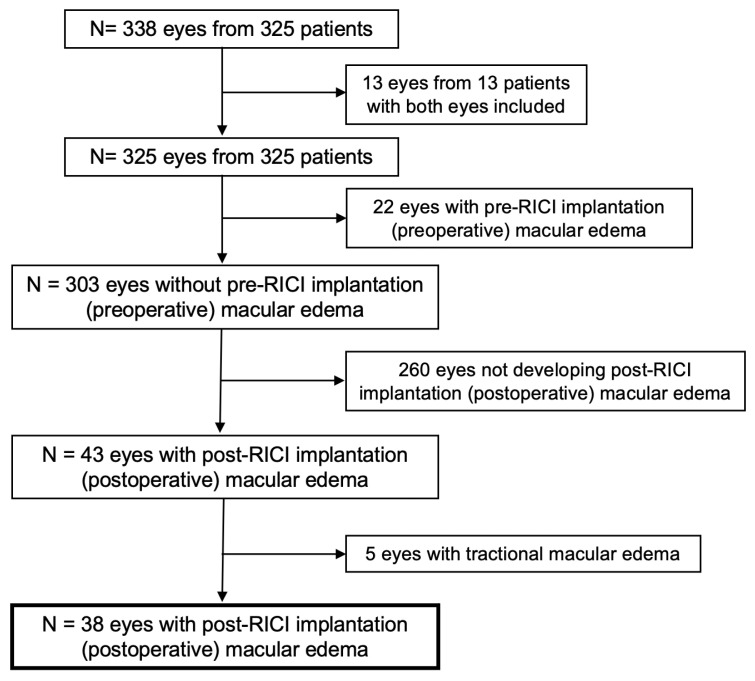
Consolidated standards of reporting trials (CONSORT)-style flowchart of included and excluded study eyes. RICI: Retropupillary iris-claw intraocular lens.

Data were collected from 338 eyes of 325 patients who underwent PPV and RICI implantation during the previously mentioned timeframe in the participating centers. In cases of bilateral surgery, only the first eye included in the study was considered for analysis, so 13 eyes were excluded from the first analysis. After RICI implantation and PPV, 65 eyes developed ME at some point during follow-up. For final analysis, 22 eyes with preoperative ME (pre-RICI implantation) and 5 eyes with tractional ME due to previous epiretinal membrane were not considered. A total number of 38 eyes with postoperative, not tractional ME, were finally analyzed (Figure 1).

Preoperative data were collected including demographics, previous medical records, ocular pathology, best-measured visual acuity (VA), biometric data, central retinal thickness (CRT) assessed by optical coherence tomography (OCT) and intraocular pressure (IOP). Indication for RICI implantation surgery was also collected and only cases of RICI implantation and associated PPV after complicated cataract surgery with no capsular support or IOL luxation were included in the analysis. Intraoperative surgical details were also reported. Collected postoperative data included slit lamp examination, VA, CRT assessed by OCT, IOP and treatment required at 1 week and 1, 3, 6 and 12 months timepoints. Postoperative ME was defined as CRT > 300 microns. Management of ME, number of intravitreal injections and type of medication administered (anti-VEGF or dexamethasone implant) data were also collected. No missing data were substituted when incomplete. The main outcome measure was ME resolution, considered when there was a complete restauration of macular anatomy assessed by OCT.

All eyes included underwent a complete standard PPV and RICI implantation under local anesthesia. The intraoperative use of triamcinolone (TA) for vitreous visualization was at the surgeon’s discretion and TA was completely cleared from the vitreous cavity after PPV completion. Postoperative treatment was common for all 14 study centers and included a topical antibiotic for at least 1 week and a 4-week treatment with topical steroids or topical NSAIDs.

Descriptive, frequency statistics and the chi-squared test were used to assess differences in the qualitative variables. Normality of the quantitative variables was examined using histograms and the Kolmogorov–Smirnov test. The values of continuous quantitative variables were expressed as mean ± standard deviation (SD) or median and interquartile range. The paired *t*-test was used to compare pre- and post-surgery changes when variables were distributed normally, and the Wilcoxon test in cases where non-parametric tests were required. Univariate Cox regression and multivariate logistic regression analysis with 95% confidence intervals (CI) were performed to assess the influence of baseline characteristics on the development of postoperative macular edema and possible confounding factors.

The cumulative probability of events (i.e., VA levels, IOP elevation, IOP-lowering treatment, ME development and ME resolution) occurring after RICI implantation are presented as survival curves using the Kaplan–Meier (KM) [21]. A bilateral type I error of 5% was established. A *p*-value < 0.05 was considered statistically significant. The statistical software SPSS 25.0 (IBM SPSS Statistics v25.0; Armonk, NY, USA, IBM Corp) was used for all statistical analysis.

## 3. Results

From 325 patients undergoing PPV and RICI implantation after complicated cataract surgery with no capsular support or IOL luxation, 11.7% (38/325) eyes developed postoperative (not tractional) macular edema. Complicated cataract surgery was the main indication for RICI implantation in 57.9% (22/38) of the cases included. Mean age of patients developing ME was 73.3 ± 11.8 and 18.4% (7/38) had DM. Preoperative baseline characteristics are described in Table 1. Mean follow-up time was 13.4 ± 9.9 months (range 1–36 months).

### 3.1. Macular Edema Development and Visual Outcomes

Performing RICI implantation after complicated cataract surgery was a significant predictive factor for developing postoperative ME (univariate analysis: OR 2.27, 95%CI 1.38–4.52, *p* = 0.02; multivariate analysis: OR 2.03, 95%CI 1.01–4.18, *p* = 0.04) (Table 2). No significant results were found for other characteristics such as performing RICI implantation after IOL luxation or baseline diabetes mellitus or uveitis.

**Figure 2:**
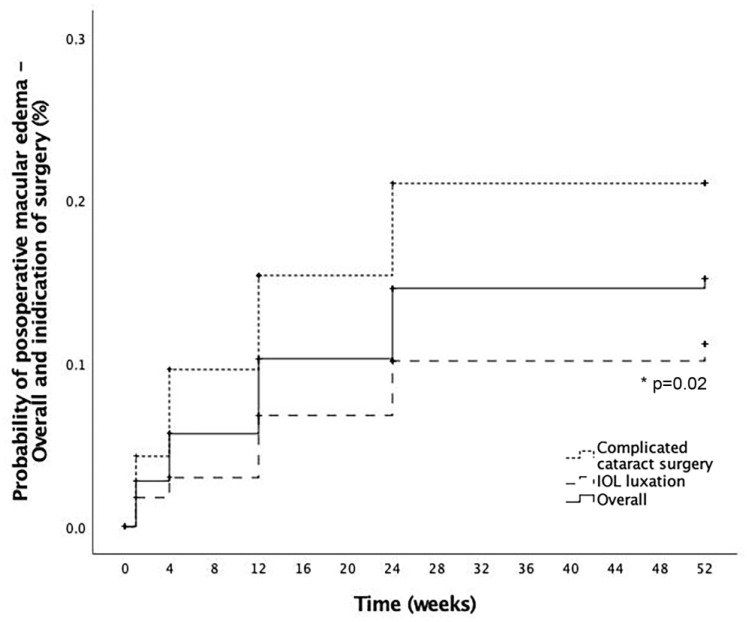
Postoperative macular edema development. Cumulative probability of postoperative macular edema (ME) in the overall cohort (solid line), eyes coming from a complicated cataract surgery (dotted line) and eyes coming from an intraocular lens (IOL) luxation (striped line). *: Statistical significance.

Mean time to ME development after RICI implantation was 11.4 ± 10.7 weeks. Mean baseline CRT was 255.6 ± 38.5 microns (μm) increasing to a peak of mean CRT change from baseline of +148.0 ± 122.2 μm at 3 months follow-up. Cumulative probability of developing postoperative macular edema in eyes with RICI implantation and not preoperative ME (*n* = 303) was 15.2% at 12 months follow-up. Cumulative probability of postoperative ME after RICI implantation in eyes with complicated cataract surgery was significantly higher than in eyes with IOL luxation (21% and 11.2%, respectively, *p* = 0.02) at 12 months (Figure 2). From the overall cohort of patients without preoperative ME (*n* = 303), no significant differences were found in the time of ME development according to the time elapsed between the complicated cataract surgery or IOL luxation and the time of RICI implantation (groups analyzed: ≤7 days; >7 days—1 month; >1 month—4 months, >4 months, *p* = 0.63). In 7.8% (3/38) of the cases, PPV and RICI implantation was performed in the same surgical session as the complicated cataract surgery.

From those patients with previous complicated cataract surgery, two groups were created depending on the time elapsed between complicated cataract surgery and RICI implantation (≤1 month, >1 month). No significant differences were observed in postoperative ME development rates (*p* = 0.93) or time to postoperative ME development (*p* = 0.61).

One of the possible independent factors of ME development could be DM. Patients with DM developing postoperative ME accounted for 18.4% (7/38) of the whole cohort. Mean time to ME development was 6.1 ± 5.6 weeks after RICI implantation. All diabetic patients had some degree of diabetic retinopathy (DR): 42.8% (3/7) mild DR, 28.6% (2/7) moderate DR and 28.6% proliferative DR, with no cases of severe DR. Previous panretinal photocoagulation (months before RICI implantation) had been performed in one patient with previous proliferative DR. Four patients 57.1% (4/7) with DM developed ME before RICI implantation at some point in their historic medical records, but did not present with ME at baseline timepoint. One patient had already received a DEX implant 4 months before RICI implantation.

**Figure 3:**
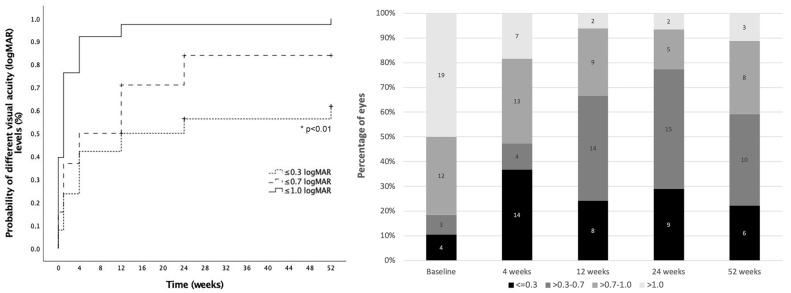
Visual acuity outcomes. Left: Cumulative probability of achieving different visual acuity (VA) levels from baseline (dotted line: VA ≤ 0.3 logMAR; striped line: VA ≤ 0.7 logMAR and solid line: VA ≤ 1.0 logMAR). Right: Distribution of eyes in each VA group at different timepoints during follow-up (black: VA ≤ 0.3 logMAR; dark grey: VA > 0.3–0.7 logMAR; medium grey: VA > 0.7–1.0 LogMAR, light grey: VA > 1.0 LogMAR). *: Statistical significance.

Mean VA (±standard deviation) at baseline was 1.4 ± 0.8 logMAR (median 1.4, IQR: 1.0) and 0.6 ± 0.4 (median 0.5, IQR: 0.4) at 3-months follow-up. Cumulative probability of VA ≤ 0.3 logMAR, VA ≤ 0.7 logMAR and VA ≤ 1.0 logMAR were 61.7%, 83.9% and 100% at 12-months follow-up, respectively. A detailed evolution of visual acuity during follow-up is presented in Figure 3.

### 3.2. Management of Macular Edema

Management of ME was performed on a case-by-case basis. Whilst some patients were followed up and received topical anti-inflammatory drops (steroids or non-steroidal anti-inflammatory drops) as the main treatment for ME, other patients underwent intravitreal injections (IT) with either anti-VEGF or a dexamethasone (DEX) implant. Acetazolamide was not used as a systemic off-label treatment for ME in this study. Following these actions, the cohort was divided in four different groups depending on the management of ME: only topical anti-inflammatory drops (31.6%, 12/38); DEX implant (50%, 19/38); anti-VEGF IT (7.9%, 3/38) or combined treatment with both DEX and anti-VEGF IT (10.5%, 4/38), as detailed in Table 3. In the DEX implant group, 57.9% (11/19) of eyes received one injection, 36.8% (7/19) received two injections and 5.3% (1/19) received three DEX implant injections. In the anti-VEGF group, 66.7% (2/3) of the patients received two injections and 33.3% (1/3) received one anti-VEGF injection. The combined treatment group was defined as eyes initially receiving anti-VEGF and switching to the DEX implant afterwards. Only one patient (25%, 1/4) was treated with one anti-VEGF and two DEX implants whilst the others received one of each. For the purpose of statistical analysis, all eyes treated with IT (anti-VEFG, DEX implant or both), which accounted for 68.4% (26/38) of the study eyes, were merged into a single group defined as the IT group.

**Figure 4:**
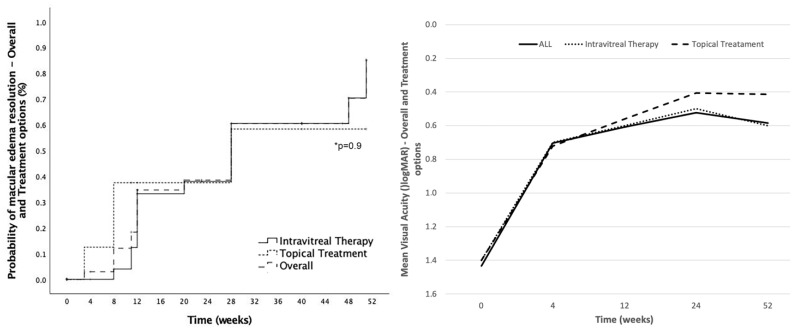
Postoperative macular edema resolution and visual acuity outcomes by treatment options. Left: Cumulative probability of postoperative macular edema (ME) resolution in the overall cohort (striped line), in eyes treated with intravitreal therapy (solid line) and in eyes treated with topical treatment (dotted line). Right: Mean visual acuity change at different timepoints during follow-up sorted by different treatment options (solid line: all eyes; dotted line: intravitreal therapy eyes; striped line: topical treatment eyes). *: Statistical significance.

Resolution of ME occurred in 50% of patients (19/38), considering resolution to be the complete restauration of macular anatomy assessed by OCT. From those, 57.9% (11/19) were in the DEX group, 21.1% (4/19) in the anti-inflammatory drops group, 10.5% (2/19) in the anti-VEGF group and 10.5% in the combined treatment group. Cumulative probability of ME resolution in the group treated with IT (DEX, anti-VEGF or both) was 85.2%, whilst in the topical anti-inflammatory drops group the probability was 58.3% at 12-month follow-up (Figure 4). Differences between groups were not significant (*p* = 0.9). Mean time to ME resolution was 19.4 ± 13.5 weeks and relapsing after resolution of ME occurred in 10.5% (4/38) of patients. From those receiving only one DEX injection, the rate of ME resolution was 54.5% (6/11). Relapsing ME occurred in three patients of the DEX group (15%, 3/19) and in one (25%, 1/4) of the combined treatment group.

With respect to the general factors associated with ME, influence of DM in postoperative ME development was specifically evaluated. Patients with postoperative ME and DM were most likely to be treated with a DEX implant (57.1%, 4/7), anti-VEGF IT (14.3%, 1/7) or a combined treatment with both a DEX implant and anti-VEGF IT (14.3%). One patient (14.3%) was treated with anti-inflammatory drops with complete resolution on ME. Mean time to ME development in these patients was 6.1 ± 5.6 weeks (range 1–12 weeks). Complete ME resolution in patients with DM occurred in 28.6% (2/7) of the study eyes, while in 42.8% (3/7) ME relapsed at some point of follow-up after resolution. Most of the relapsing cases of ME in this study were patients with DM (75%, 3/4). In two cases (28.6%, 2/7), ME did not resolve during follow-up. No association between degrees of DR and ME resolution was found. Complete resolution of ME occurred in one patient with mild DR and one patient with proliferative DR. Relapsing ME was found in two eyes with moderate DR and one eye with mild DR.

**Figure 5:**
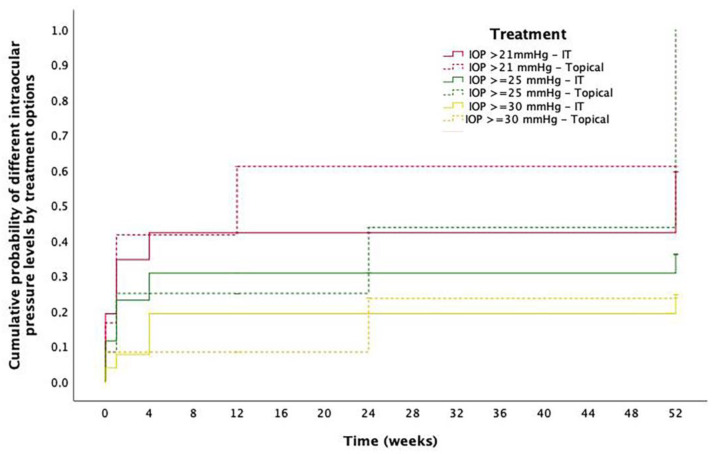
Intraocular pressure outcomes. Cumulative probability of different levels of intraocular pressure (IOP) at different timepoints in eyes with postoperative macular edema sorted by treatment option. Red solid line: Cumulative probability IOP > 21 mmHg in eyes treated with intravitreal therapy (IT); Red striped line: Cumulative probability IOP > 21 mmHg in eyes treated with topical treatment; Green solid line: Cumulative probability IOP ≥ 25 mmHg in eyes treated with IT; Green striped line: Cumulative probability IOP ≥ 25 mmHg in eyes treated with topical treatment; Yellow solid line: Cumulative probability IOP ≥ 30 mmHg in eyes treated with IT; Yellow striped line: Cumulative probability IOP ≥ 30 mmHg in eyes treated with topical treatment.

As described, the DEX implant was the most common treatment for ME after RICI implantation. No migrations to anterior chamber were described nor cases of endophthalmitis in the IT group. Mean preoperative IOP in the study eyes was 17.4 ± 7.4 mmHg and 16.4 ± 6.8 mmHg after 1-month follow-up. Cumulative probability of IOP > 21 mmHg, ≥ 25 mmHg and ≥ 30 mmHg at 12-months follow-up was 59.6%, 36.1% and 24.6%, respectively, for the IT group and 61.1%, 43.8% and 0.24% for the topical treatment group. Differences between groups were not significant at any IOP level. A detailed description of IOP evolution in these patients is described in Figure 5. Two eyes treated with a DEX implant underwent a filtrating surgery at some point during follow-up. Both patients had been preoperatively diagnosed with glaucoma.

### 3.3. Other Complications

Disenclavation of one of the IOL claws occurred in two cases (5.3%, 2/38) and a secondary surgery was performed for IOL relocation. No other complications were described.

## 4. Discussion

The current study evaluates possible predictive factors, outcomes and management of thirty-eight eyes that developed postoperative ME after RICI implantation in a large multicenter series of aphakic eyes. All patients underwent RICI implantation and PPV after complicated cataract surgery without capsular support or IOL luxation. To date, scarce data are available in the literature about the real incidence and possible predictive factors of postoperative ME development after RICI implantation [4,5,6,7,22] and, similarly, there is no consensus about the optimal postoperative management of this condition. As far as we know, this is the first analysis specifically directed to investigate predictive factors, management and clinical outcomes of postoperative ME after RICI implantation. These data might be relevant for patient counselling and decision making in daily clinical practice.

Postoperative ME was one of the main complications of RICI implantation in our previously published report of this multicenter national cohort [4], resulting in a cumulative probability of 20.5% of ME development. This figure is substantially higher than the ones presented in previous reports [2,3,5,6,7,23], which may be explained by different factors such as differences in the surgical technique of RICI implantation (in our study all patients underwent PPV) or the cause of aphakia, with the only indications for RICI implantation considered in our series being complicated cataract surgery with no capsular support or IOL luxation. In the evaluation of possible preoperative factors which may predispose to the development of postoperative ME, the only significant characteristic found was performing RICI implantation after a complicated cataract surgery with no capsular support (OR: 2.27; 95% CI: 1.38–4.52, *p* = 0.02). This result is consistent with previous reports where posterior capsule rupture with vitreous loss considered to be a risk factor for developing postoperative ME [24]. The time elapsed between the complicated cataract surgery and the RICI implantation surgery could potentially be a driving factor for ME development. However, in our series, no statistically significant differences were found between the rate of ME development or the time to ME development in the subgroup analysis by the time elapsed between the complicated cataract surgery and the time of RICI implantation.

With regards to diabetes mellitus, which was considered as a potential risk factor for ME, the condition did not predispose to postoperative ME development in the present study (OR: 1.35; 95% CI: 0.55–3.28, *p* = 0.51). In our series, diabetic patients had more probability of developing a relapse of ME after resolution than non-diabetic patients and 75% of overall relapsing ME occurred in diabetic patients. One of the main issues related to ME development in patients with DM is how to differentiate between postoperative ME and diabetic ME appearing after surgery. Some studies have tried to describe this difference using particular OCT parameters, but currently there is not enough evidence to build this distinction by OCT and, even in these cases, an FFA may be still inconclusive [25]. The time to ME development or the duration of DM might be key differential factors, but there was an important variability in these features in our cohort to enable the drawing of any conclusions. However, even if our results did not confirm these points, diabetic patients seemed to have a shorter time to ME development after RICI implantation than non-diabetic patients.

Management of ME after surgery remains controversial and, to date, there are no generally accepted, standardized treatment guidelines. However, sometimes postoperative ME can spontaneously resolve, and in other cases may persist causing permanent damage to the retina with associated visual loss and its management may be challenging for clinicians. Different treatment strategies have been suggested, with the staggered regimen being one of the most accepted options. However, the evaluation of the efficiency of postoperative ME treatments is always conditioned by the possibility of spontaneous resolution which might be a major confounder in these cases [26].

Following this staggered regimen, first-line treatment would consist of the employment of topical NSAIDs with or without the association of topical steroids or acetazolamide (off-label), with the aim of controlling inflammation which has been described as one of the main pathophysiological pathways for ME development after surgery. NSAIDs have been proven to reduce the incidence of postoperative ME when used as a preventive treatment. However, different results have been reported with respect of topical NSAIDs efficiency in ME resolution [27]. In our study, 58.3% of the patients treated with topical NSAIDs had complete resolution of ME. These results suggest that this treatment alone for ME after RICI implantation may not be sufficient. However, we have to consider a hypothetical selection bias, as this first-line treatment may have been used in cases of mild ME with preserved VA or residual ME, keeping intravitreal treatment for refractory cases or for more severe cases.

Second-line treatments include periocular and intravitreal injections. Sub-Tenon’s or Retrobulbar steroid injections (i.e., triamcinolone) have been demonstrated to be effective in persistent ME refractory to classic anti-inflammatory topical treatment [18]. Intravitreal injections of anti-VEGF are also considered as a possible treatment for postoperative ME with some studies describing positive results and reduction in CRT [28]. In our study, 7.9% (3/38) of the eyes were only treated with anti-VEGF therapy. Moreover, 10.5% (4/38) of patients, who were initially treated with anti-VEGF, at some point of follow-up switched treatment to the DEX implant, suggesting that anti-VEGF might not be enough for ME resolution in cases of postoperative ME after RICI implantation. It seems that the preferred option for ME initially treated with anti-VEGF that persists or relapses is switching to a DEX implant. Indeed, the injection of a DEX implant was the most commonly used treatment for postoperative ME after RICI implantation in our study.

Intravitreal therapy was used in 68.4% (26/38) of the eyes with postoperative ME. The DEX implant was the elected treatment in 50% (19/38) of the cases. As demonstrated in EPISODE-1 and -2 studies, the DEX implant is an effective and safe treatment for postsurgical ME [15,16]. In our cohort, we described a cumulative probability of postoperative ME resolution after IT (most of them were patients treated with DEX implant) of 85.2%. These results, in comparison with the ones obtained with the topical treatment (58.3%), have to be contextualized. As previously mentioned, a treatment selection bias may have been applied as a topical treatment could have been used in milder ME cases. However, despite being milder cases, the lower final rate of complete resolution of postoperative ME in these eyes may be a consequence of tolerating small amounts of residual ME with preserved VA in some of the study eyes that received a topical treatment during follow-up.

The use of different success criteria in the published literature at the time of evaluating the effectiveness of postoperative ME treatments (some studies use the number of letters gain, while others consider the anatomic criteria) makes it difficult to contextualize our results with other published reports. In our study, VA was not considered as a main success criterion although VA gain was observed after ME resolution. Instead, anatomic criteria were considered for both success evaluation and retreatment decision making. With regards to repeated injections, 57.9% (11/19) of eyes required one DEX injection and 42.1% (8/19) of eyes required more than one DEX implant at some point of follow-up for persistent ME. These figures are slightly higher than the ones reported by Bellocq et al., where 37% of patients included in the study only required one DEX implant during 1 year follow-up for resolution of postsurgical ME [15]. In previous reports supporting the effectiveness of the DEX implant for the treatment of persistent postoperative ME, the inflammatory nature of the edema and the lack of comparative data between anti-VEGF and DEX implants in these particular cases may have been the reasons for decision making in these studies [10,11,15,16,28,29,30,31,32,33]. Moreover, and as shown in our study, four patients undergoing anti-VEGF first were switched to a DEX implant (combined treatment group), suggesting that the DEX implant might be more effective in cases of RICI implantation and PPV. The fact that all studied patients were vitrectomized could have played a role in effectiveness of the anti-VEGF injection, which has been related in some reports to less anatomical and functional improvements in vitrectomized eyes [34]. Despite the fact that iris-claw IOLs for aphakia management are a relative contraindication for DEX implants and implant migration to the anterior chamber has been detailed in the literature [35], no cases with this complication were observed in our study cohort. We believe that, taking into account the suboptimal response shown in our series to other treatment alternatives, the benefits of the DEX implant outweigh the risks, although the limited follow-up period prevents making robust statements in this field beyond 12 months. Standard complications associated with the DEX intravitreal implant were not different than the figures described in our previous study [4], and were not significantly higher with respect to eyes treated only with topical anti-inflammatory drops. With the provided data, we suggest that the DEX implant appears as a good, effective and safe option for the treatment of postoperative ME associated with RICI, with the precaution of informing patients about the potential risk of implant migration to the anterior chamber in the risk–benefit discussions.

The limitations of the study are, on the one hand, the retrospective nature of the study which means that treatment options for postoperative ME after RICI implantation were not randomized and, consequently, the groups may not be comparable. However, conducting a prospective multicentric study on this topic would be challenging, as no pre-specified protocol is universally adopted for the management of post-operative ME, particularly in complicated cataract cases such as the ones included in this series. Moreover, the fact of being a multicenter study, although offering the possibility of acquiring a large series of eyes, may have also induced some variability in the treatment decision-making and results. However, this study offers real-life clinical data about the characteristics of the postoperative edema, the patient and the clinician preferences, since the treatment options were personalized. The lack of prospective studies in this field prevents the identification of the optimal treatment pathway for this condition, and, in this scenario, this study shows the results of different treatment options in routine clinical care in a multicenter setting.

In summary, this study identifies potential predictive features for postoperative ME development in a large national series of aphakic eyes. In particular, the indication of RICI implantation after a complicated cataract surgery with no capsular support was the greatest risk factor identified for postoperative ME development. This study also provides relevant data about the clinician’s preferred therapy of choice for ME management and the clinical outcomes obtained with the different options in this selected cohort. The information provided in this study may help clinicians at the time of treatment decision in cases of aphakic patients managed with the RICI technique in routine clinical care.

## Figures and Tables

**Table 1:** Preoperative Baseline Characteristics of study eyes.

	Eyes with Postoperative Macular Edema				Eyes without Postoperative Macular Edema			
	Total	Complicated Cataract Surgery	IOL Luxation	*p*-Value	Total	Complicated Cataract Surgery	IOL Luxation	*p*-Value
	38 eyes	57.9% (22/38)	42.1% (16/38)	-	260 eyes	39.6% (103/260)	60.4% (157/260)	
Gender (*Male %*)	68.4 (26/38)	72.7 (16/22)	62.5 (10/16)	0.51	56.1 (146/260)	49.5 (51/103)	60.5 (95/157)	0.08
Age (years)				0.17				0.28
(*Mean ± SD*)	73.3 ± 11.8	74.1 ± 10.9	79.0 ± 11.6		73.1 ± 1.2	74.0 ± 13.7	72.1 ± 15.4	
(*Median; IQR*)	76.0; 9.5	78.0; 12	82; 7.5		76; 16	77.5; 12.7	76; 16	
Preoperative VA (logMAR)	1.4 ± 0.8	1.7 ± 0.7	1.2 ± 0.8	0.06	1.19 ± 0.7	1.3 ± 1.3	1.1 ± 0.7	0.02
	1.4; 1.0	1.8; 1	1.0; 1.3		1; 1.3	1.3;1.5	1; 1.3	
Preoperative IOP (mmHg)	17.4 ± 7.4	16.8 ± 5.0	18.2 ± 10.3	0.60	17.5 ± 6.9	16.7 ± 1.3	17.9 ± 6.7	0.21
	16; 7.5	16; 6	16.0; 8		16; 6	16; 5	16; 6	
Axial length (mm)	23.9 ± 2.1	24.0 ± 2.4 23.3; 1.5	23.6 ± 1.4 23.1; 1.3	0.71	24.0 ± 2.0 23.6; 2.4	23.1 ± 1 0.3 22.9; 1.4	24.5 ± 2.2 24.1; 2.6	<0.01
Diabetes mellitus (%)	18.4 (7/38)	18.2 (4/22)	18.7 (3/16)	0.89	14.2 (37/260)	16.5 (17/103)	12.7 (20/157)	0.34
Diabetic retinopathy (%)	18.4 (7/38)	18.2 (4/22)	18.7 (3/16)		6.9 (18/260)	9.7 (10/103)	5.1 (8/157)	
-Non-proliferative:								
-Mild (%)	42.8 (3/7)	50 (2/4)	33.3 (1/3)		77.8 (14/18)	80 (8/10)	75 (6/8)	
-Moderate (%)	28.6 (2/7)	25 (1/4)	33.3 (1/3)		22.2 (4/18)	20 (2/10)	25 (2/8)	
-Severe (%)	0 (0/7)	-	-		0 (0/18)	0 (0/10)	0 (0/8)	
-Proliferative (%)	28.6 (2/7)	25 (1/4)	33.3 (1/3)		0 (0/18)	0 (0/10)	0 (0/8)	
Central Retinal Thickness (μm)				0.74				0.90
(*Mean ± SD*)	273.4 ± 58.0	276.27 ± 65.8	263.0 ± 6.1		241.0 ± 65.5	242.2 ± 1.3	240.2 ± 78.5	
(*Median; IQR*)	263.5; 58.5	267; 87	260; 5.5		249; 40	248; 35	251; 43.7	
Pseudoexfoliation (%)	21.0 (8/38)	18.2 (4/22)	25 (4/16)	0.67	28.5 (74/260)	26.2 (27/103)	29.9 (47/157)	0.47
Glaucoma (%)	21.0 (8/38)	27.3 (6/22)	12.5 (2/16)	0.26	20 (52/260)	17.5 (18/103)	21.6 (34/157)	0.42
High Myopia (%)	5.3 (2/38)	4.5 (1/22)	6.2 (1/16)	0.82	12.7 (33/260)	5.8 (6/103)	17.2 (27/157)	<0.01
Uveitis (%)	2.6 (1/38)	4.5 (1/22)	-	-	3.5 (9/260)	4.8 (5/103)	2.5 (4/157)	0.28
Branch Retinal Vein Occlusion (%)	2.6 (1/38)	4.5 (1/22)	-	-	0.8 (2/260)	0.9 (1/103)	0.6 (1/157)	0.76

VA: Visual acuity; IOL: Intraocular lens; SD: Standard deviation; IQR: Interquartile range; IOP: Intraocular pressure.

**Table 2:** Univariate and multivariate analysis of predictive factors of postoperative macular edema.

	Postoperative Macular Edema (n = 38) (%)	No Postoperative Macular Edema (n = 260) (%)	Univariate Analysis			Multivariate Analysis		
			*p*-Value	OR	95% CI	*p*-Value	OR	95% CI
Complicated cataract surgery	57.9 (22/38)	37.7 (100/260)	0.02	2.27	1.38–4.52	0.04	2.03	1.01–4.18
Pseudoexfoliation	21.1 (8/38)	29.1 (77/260)	0.30	0.65	0.29–1.48	0.29	0.61	0.24–1.53
Diabetes Mellitus	18.4 (7/38)	14.3 (38/260)	0.51	1.35	0.55–3.28	0.52	1.35	0.54–3.39
Glaucoma	21.6 (8/38)	20.3 (53/260)	0.85	1.08	0.47–2.51	0.75	1.16	0.47–2.87
High myopia	5.3 (2/38)	14.3 (38/260)	0.12	0.33	0.08–1.44	0.22	0.39	0.09–1.74
Uveitis	2.6 (1/38)	3.8 (9/260)	0.72	0.69	0.08–5.59	0.65	0.60	0.07–5.12

OR: Odds Ratio; CI: Confidence Interval.

**Table 3:** Macular edema treatment options.

Treatment	Total	Dexamethasone	Anti-VEGF	Mixed Treatment		Topical Treatment
Number of patients	38	50% (19/38)	7.9% (3/38)	10.5% (4/38)		31.6% (12/38)
Number of intravitreal injections per patient	1 injection	57.9% (11/19)	33.3% (1/3)	1 Anti-VEGF + 1 DEX implant	75% (3/4)	NSAIDs new
	2 injections	36.8% (7/19)	66.6%% (2/3)	-		NSAIDs classic
	3 injections	5.3% (1/19)	-	1 Anti-VEGF + 2 DEX implants	25% (1/4)	Topical steroids

Anti-VEGF: intravitreal anti-vascular endothelial growth factor; DEX: Dexamethasone; NSAIDS: Non-steroidal anti-inflammatory drugs.

## Data Availability

The data presented in this study are available on request from the corresponding author. The data are not publicly available due to privacy.

## Appendix A. Spanish Multicenter Iris-Claw IOL Study Group (Sorted by Participant Centers, Principal Investigator and in Alphabetical Order)

- Hospital Clínic de Barcelona, Barcelona (Coordinating center): Javier Zarranz-Ventura, Alfredo Adán, Carolina Bernal-Morales, Manuel J. Navarro-Angulo
- Althaia Xarxa Assistencial Universitària de Manresa: Eduard Pedemonte-Sarrias, Fátima Sánchez-Aparicio
- Hospital Clínico San Carlos, Madrid: Alicia Valverde
- Hospital Clínico Universitario Lozano Blesa, Zaragoza, Spain: Javier Ascaso, Olivia Esteban
- Hospital Donostia, San Sebastián: Cristina Irigoyen, Luis Ansa, Leire Juaristi, Miguel Ruiz-Miguel
- Hospital La Arruzafa, Córdoba: Juan Manuel Cubero-Parra, Juan Manuel Laborda, Consuelo Spínola-Muñoz
- Hospital Povisa, Vigo: Daniel Velazquez-Villoria
- Hospital Universitario de Basurto, Bilbao: Estibaliz Ispizua-Mendivil, M. Paz Mendivil-Soto
- Hospital Universitario Mútua Terrassa, Terrassa: Marina Barraso-Rodrigo, Sophia Bennis
- Hospital Universitario Ramon y Cajal, Madrid: Diego Ruiz-Casas, Marta Gómez-Mariscal, Julio José González-López
- Hospital Universitario Valme, Sevilla: Adrián Hernández-Martínez
- Hospital Universitario Virgen Macarena, Sevilla: Isabel Relimpio, Enrique Rodríguez-de-la-Rua, Ainhoa Roselló
- Hospital Universitario Virgen del Rocío, Sevilla: Mariano Rodriguez-Maqueda, José Luis Sánchez-Vicente, Amparo Toro-Fernández, Lorenzo Trujillo-Berraquero
- Instituto Oftalmológico La Esperanza, HM La Esperanza, Santiago de Compostela & Servicio de Oftalmología, Complejo Hospitalario Universitario, A Coruña: Joaquín Marticorena
